# Willingness to Share Internet Use Data for Research on Early Disease Detection: Cross-Sectional Survey

**DOI:** 10.2196/85637

**Published:** 2026-03-25

**Authors:** Christina Derksen, Mel Ramasawmy, Sanjula Arora, Wynsee Lau, Suzanne E Scott

**Affiliations:** 1 Wolfson Institute of Population Health, Queen Mary University of London, Charterhouse Square, London, EC1M6BQ, United Kingdom, 44 2078823850; 2 Faculty of Medicine, Imperial College London, London, United Kingdom

**Keywords:** early detection, internet use data, willingness to share online data, public perceptions, data privacy

## Abstract

**Background:**

Preliminary research has suggested that internet use data could offer digital signals of early disease and has the potential to facilitate early detection and improve patient outcomes. However, there are significant challenges in linking individual-level internet use data with health outcomes. One key aspect is that the public might not be willing to share data for research or that selective data sharing might create bias in datasets and increase inequalities.

**Objective:**

Our study aimed to investigate the willingness of the public to share their internet use data for medical research and to identify key criteria that affect willingness to share.

**Methods:**

We conducted a web-based, cross-sectional online survey with 2390 UK adults with and without a history of cancer, heart disease, and depression using quota sampling. Participants were randomly assigned to explore willingness to share different types of internet use data for 1 of 3 health conditions (cancer, heart disease, and depression) and for provision of a pictorial example of internet use data. Logistic regression analysis (α=.05) for each condition was used to determine key factors of willingness to share, including sociodemographics and attitudes toward sharing. Open-ended comments regarding facilitators of sharing and concerns were analyzed thematically.

**Results:**

Willingness to share internet use data was high across conditions (74%‐77%, 95% CI 70.5%‐80.3%), especially for health app data (73%‐76%, 95% CI 69.8%‐79.1%). The pictorial example of browsing history did not affect willingness to share. For all conditions, factors consistently associated with willingness to share were perceived benefits (odds ratios [ORs] 5.692‐8.850; all *P*<.001) and concerns (ORs 0.343‐0.432; all *P*<.001). Key concerns were data privacy, potential for misuse, and lack of relevance. Suggestions to increase willingness to share included contributing to society and research, data security assurances, clarification of research purposes, and monetary incentives. Familiarity with internet use data was related to lower willingness to share for heart disease detection (OR 0.740, 95% CI 0.561‐0.976). Asian ethnicity was associated with lower willingness to share internet use for cancer detection (OR 0.234, 95% CI 0.076‐0.723). Younger age (OR 0.975, 95% CI 0.951‐0.999) and male gender (OR 2.615, 95% CI 1.511‐4.526) were associated with higher willingness to share data for depression detection.

**Conclusions:**

This first large-scale assessment of public willingness to share internet use data for early disease detection adds novel insights by comparing conditions and examining sociodemographic factors alongside perceived benefits and risks. It highlights that understanding of internet data is limited yet willingness to share for research is high. Clear communication of benefits, strong privacy protections, and incentives may increase participation and reduce bias. The findings inform consent design, targeted outreach to underrepresented groups, and data governance for safe use of personal digital data. Future research should focus on improving public communication, particularly among less willing groups at risk of inequality.

## Introduction

Across many health conditions, early diagnosis is crucial for successful treatment and long-term outcomes, potentially impacting survival and health care costs [1-3]. Early diagnosis depends on detection of signs and symptoms, presentation to health care professionals, and timely diagnostic tests. However, patients often present to health care weeks or months after the onset of symptoms, and a key reason for this delay is the time taken to interpret symptoms as potentially serious [4,5]. Thus, the understanding of timing of symptom perception, symptom interpretation, and help-seeking behavior is vital in efforts to encourage early diagnosis.

“Digital footprints” capture huge amounts information, including online activity, in-store purchases, wearable device data, and social media engagement [6]. These digital data may provide insights into symptoms, changes in behavior, and the decision to seek help [7,8], and using these data may tackle current research limitations in this area. To date, researchers studying time to presentation have relied on retrospective self-report from patients recently diagnosed with the condition under investigation [9]. These data are subject to recall bias and could be affected by hindsight from the recent diagnosis [10,11].

In the United Kingdom, there are almost 50 million health-related searches per year using Google [12]. Internet use data include internet search history, calendar data, bookmarks, contacts, emails, photos, reminders, profiles, and documents stored online. Given the richness of these data, there is growing interest to explore the use of internet search data and other digital footprints as digital signals of serious disease. Preliminary research has begun to examine whether data on internet search history could be used to predict diagnosis of cancers and mental health conditions, with promising results. Changes in the frequency, content, and tone of online searches were evident up to 60 days before suicide attempts or general practitioner referral for gynecological cancers, up to 21 weeks before pancreatic cancer diagnosis, and up to 1 year before psychiatric hospitalization [13-16].

Despite the promise of the digital signals, there are multiple questions that need to be explored regarding the acceptability of this kind of research for patients and the public, especially if research involves sharing internet use data on an individual level and linking with future health outcomes such as a cancer diagnosis. Previous studies have mixed results regarding people’s willingness to share different types of digital data over “traditional” types of health data such as biomedical samples or questionnaire data [17,18]. It is unclear if internet users are aware that these data exist, where they are stored, and how they could be used in research or if effective ways of explaining these issues to potential participants exist. This is key to informed consent.

Willingness to share might depend on several factors, including the type of data being shared, the way internet data are explained, the purpose of the research, privacy concerns, and sociodemographic factors [19]. If some groups are less willing to provide internet use data, findings from future research might not be representative and could thus perpetuate health inequalities. For example, gender, age, ethnicity, or socioeconomic status such as employment might influence willingness to share internet use data and other types of data such as location data or measures from health and fitness apps [19-21]. Further, the type of condition being studied and the relevance to an individual (eg, through personal history or experiences of friends and family being affected by a mental or physical health condition) can influence willingness to participate in medical research [22], yet it is unknown if this applies to willingness to share internet use data.

This study therefore investigated public perspectives on the use of internet use data for medical research to identify key criteria that could enhance acceptability and avoid inequalities in engagement. Our research questions were exploratory:

1. How willing are the public to share different types of internet use data for early diagnosis research?
2. Does willingness to share internet use data vary for different mental and physical health conditions?
3. Does the way internet use data is explained impact willingness to share internet use data?
4. Are sociodemographic characteristics, personal history of different mental and physical health conditions, familiarity with internet use data, and attitudes toward data sharing associated with willingness to share internet use data?

## Methods

### Study Design

We used a cross-sectional design to conduct an online survey on willingness to share different types of internet use data. We randomly assigned participants using an automated algorithm based on 2 factors: (1) the medical condition about which participants are asked whether they would be willing to share their data (cancer, heart disease, and depression) and (2) the way internet use data was explained (written text plus a screenshot as a pictorial example of internet use data vs written text only). Cancer, depression, and heart disease were selected, as these are common conditions for which early diagnosis is key and there is emerging evidence for use of digital data in prediction models [13-16]. We reported the study in line with Journal Article Reporting Standards Quantitative Research Design (JARS-Quant) standards [23].

### Ethical Considerations

Ethical approval for research with human subjects was granted by the Queen Mary Ethics of Research Committee (reference number QME25.0924). Participants received the participant information sheet for download at the beginning of the survey and provided informed consent for participation and data sharing before they could access the survey. All data were collected anonymously. In line with Dynata’s company policies, participants received tokens through the loyalty programs set up through Dynata partnerships automatically upon survey completion, which could be redeemed for vouchers or gifts. Incentives were valued at approximately £0.75 (US $1.00). No identification of individual participants in any images of the manuscript or appendices is possible.

### Participants and Procedure

Participants were adult members of the public (18 years or older), both with and without a personal history of cancer, heart disease, or depression. The survey was hosted on the online platform Qualtrics. Participants were recruited through Dynata from participants who had previously signed up to an online panel from July 1, 2025, to July 23, 2025. As the panel was English-speaking and conducted initial checks with new participants, we assumed all participants would be proficient in the English language. Before full recruitment, the survey was pilot tested with patient and public representatives (see the following sections). To ensure data quality, it was then released to 10% of the final population to prevent technical problems and unclear questions before full roll-out.

The survey was administered using quota sampling based on gender (approximately 50% male and 50% female), age (approximately 50% of the population over the age of 50 years), income (approximately 50% with disposable household income levels <£35,000), and ethnicity (maximum 85% of the population from a White ethnic group). A minimum of 300 participants with a history of cancer, heart disease, or depression was sought. We chose to sample for age to increase the proportion of older participants, who have a higher incidence of cancer and cardiovascular disease. Online survey panels often have a disproportionately higher share of younger respondents [24], and we anticipated that younger participants would be more likely to take part in a survey on the topic of internet use and data.

### Measures

The survey assessed willingness to share data for early diagnosis research, attitudes toward data sharing, familiarity about internet use data, and sociodemographics. All items and the pictorial example can be found in Multimedia Appendix 1.

#### Willingness to Share Internet Use Data for Early Diagnosis Research and Attitudes Toward Data Sharing

The main outcome measure was willingness to share data, assessed as a single item adapted from Hirst et al [19] with 4 answer options (definitely yes to definitely no). Where appropriate, this was recoded to a binary variable (yes, no) for analyses. The same question was used to assess willingness to share specific types of internet use data (eg, internet search history or online purchases), with an option to choose “Not applicable (I do not use this).”

Open-ended questions were used to ask participants what would increase their willingness to share internet use data, as well as their concerns about data sharing.

Attitudes toward data sharing were assessed as perceived benefits and concerns or worries using an adaptation of scales by Sanderson et al [24] and Seltzer et al [18]. We used matrix questions to include perceived benefits and concerns and calculated mean scores (Cronbach *α*=0.96 for perceived benefits and 0.94 for concerns).

#### Familiarity With Internet Use

We assessed participants’ self-reported familiarity with internet use data by adapting the scale by Hirst et al [19] on awareness about General Data Protection Regulation (GDPR). We asked whether participants had heard about internet use data before. Objective knowledge was assessed by asking what participants thought was part of internet use data from a list of examples (eg, internet search history as example of internet use data or content of emails as distractor). Correct answers were counted to calculate a knowledge score (range 1‐10).

#### Sociodemographics and Current Internet Use

For sociodemographic characteristics, we assessed age, gender, educational level, employment, marital status, ethnicity, and preferred language. Items were adapted from the DISTINCT project [25]. We assessed disposable household income (<£35,000 or >£35,000) and postcodes (to determine levels of deprivation via English indices of deprivation 2019 [26], Postcode Deprivation Finder 2019 [27], and Multiple Deprivation Measures 2017 Lookup Tool [28]). Finally, we measured self-reported history of cancer, cardiovascular disease, and depression and whether their relatives had a history of these conditions. Answer options were adapted from the Health Survey for England, 2021 [29].

To assess current internet and online service use, we used 2 questions from the UK Digital Landscape survey [30] about frequency and purpose of internet use.

### Data Preparation

There were no missing data in the study variables such as willingness to share internet use data, self-reported familiarity with internet use data, and perceived benefits or concerns, as items were set to mandatory. In the sociodemographic data, <1% were missing, except for disposable household income (2.3%) and deprivation quintile (7.8%). Online surveys are open to fraudulent responses [31,32]; as a precaution, we introduced a number of steps to ensure credibility of the data. An attention check was added halfway through the survey, asking participants to select “strongly agree” with answer options appearing in a random order. Participants were excluded if they failed the attention check. We also excluded participants who were flagged by Qualtrics as both likely bot *and* fraudulent respondent (Recaptcha Score<0.5 and Fraud Score>30) or as duplicate (flagged by Qualtrics or on inspection of the data), as well as participants who answered the survey in less than 2 minutes, which was considered to be insufficient time to read through the participant information sheet and questionnaire items. Finally, we excluded participants with the common placeholder “lorem ipsum” text or questionable open answers (eg, “Banana”) on both open-ended questions.

### Data Analysis

We descriptively report differences in the willingness to share internet use data for early diagnosis research using percentages of participants indicating they would be willing to share. We conducted *χ*^2^ tests for differences in proportions willing to share internet use data between medical conditions, the way internet use data were explained, and history of the condition. Logistic regression analysis was used to determine how sociodemographic characteristics, perceived benefits and concerns, knowledge, and familiarity with internet use data affect willingness to share internet use data, coded as a binary outcome. Separate regression models were run for willingness to share internet use data for each medical condition (cancer, heart disease, depression).

Responses to open-ended questions were analyzed using a thematic content analysis approach and reported alongside how willing participants were to share internet use data. Categories were developed by two authors (CD and WL) and discussed with the research team including patient and public involvement (PPI) representatives. Answers not in English (one Romanian and one Polish) were translated using Google Translate, as translated content fit the questions, showing that participants understood English but answered in a different preferred language.

The full syntax and the final datasets can be found on Open Science Framework (OSF) [33].

### Power Analysis

Based on previous research [19], we made the conservative assumption that willingness to share internet use data would likely vary between 45% and 55% of participants being willing to share. For an a priori sample size calculation, we therefore assumed a difference of 0.1 in probabilities of being willing to share data or not for a binary factor (0.55 in one group, 0.45 in the other; odds ratio [OR] 1.49). To reach a power of 80% given an α-error level of .05, we determined a sample size of 786 participants per regression. As logistic regression analysis was repeated for each condition, we aimed for a sample size of 2358 participants (3 × 786). However, the sample sizes for the logistic regression analyses were lower than the targeted sample size, so analyses were repeated without the variable with the most missing data (level of deprivation; see the following sections) for sensitivity analysis.

### Patient and Public Involvement

We presented initial ideas for survey items to 3 PPI representatives who were older than 55 years and from a minority background. They were recruited from the Centre for Cancer Screening, Prevention and Early Diagnosis PPI pool at Queen Mary University of London, which is a database of more than 150 people with an interest in contributing to research. They endorsed the research but asked to add questions about emotions (fear and embarrassment) to the items on attitudes toward data sharing. The group asked that the participant information sheet emphasized that data sharing was not required for participation in this study and to change the wording of some items to facilitate understanding. Following these changes, we piloted the survey with 2 PPI representatives in think-aloud sessions to ensure face validity [34]. The PPI group was also involved in interpreting the results and discussing implications for future research and practice.

## Results

### Participant Characteristics

Of the 2864 participants who gave informed consent and completed the survey, 474 were excluded: 365 failed the attention check, 32 were flagged as both likely bot and fraudulent respondent, 64 were duplicate respondents, 6 completed the survey in less than 2 minutes, and 7 gave questionable open answers. Hence, 2390 participants with a mean age of 50.2 (SD 16.86, range 18‐90) years were included in the analyses. Participant characteristics are provided in Table 1.

**Table 1.** Participant characteristics from a cross-sectional survey in 2025 conducted with UK participants recruited through an online survey panel (N=2390).

Characteristic		Results, n (%)
Gender		
Man		1250 (52.3)
Woman		1135 (47.5)
Nonbinary		4 (0.2)
Prefer not to say		1 (<0.1)
Employment status		
Employed (including self-employed)		1433 (60)
Not employed		352 (14.7)
Retired		592 (24.8)
Prefer not to say		13 (0.5)
Highest educational qualifications		
No formal qualifications		73 (3.1)
GCSEs^a^ or equivalent		408 (17.1)
AS^b^, A level or equivalent		296 (12.4)
NVQ^c^ up to level 3 or equivalent		256 (10.7)
Qualification at degree level or above		1250 (52.3)
Apprenticeship		51 (2.1)
Other or equivalent unknown		52 (2.2)
Prefer not to say		4 (0.2)
Disposable household income (£)		
<35,000		1206 (50.5)
>35,000		1127 (47.2)
Prefer not to say		57 (2.3)
Deprivation quintile based on postcode		
1 (most deprived)		464 (19.4)
2		522 (21.8)
3		425 (17.8)
4		385 (16.1)
5 (least deprived)		408 (17.1)
Missing		186 (7.8)
Marital status		
Single		662 (27.7)
Married or in legal relationship		1368 (57.2)
Widowed, separated or divorced		348 (14.6)
Prefer not to say		12 (0.5)
Ethnicity		
White		1978 (82.8)
Asian or Asian British		127 (5.3)
Black, Black British, Caribbean or African		216 (9)
Mixed or multiple ethnic groups		51 (2.1)
Other		8 (0.3)
Prefer not to say		10 (0.4)
Preferred language		
English		2376 (99.4)
Other		12 (0.5)
Prefer not to say		2 (0.1)
Personal history of medical conditions		
Cancer		398 (16.7)
Heart disease or vascular problems		422 (17.7)
Depression		745 (31.2)
None of the above		1047 (43.8)
Not sure/not formally diagnosed		126 (5.3)
Prefer not to say		16 (0.7)
Relatives’ history of medical conditions		
Cancer		656 (27.4)
Heart disease or vascular problems		633 (26.5)
Depression		535 (22.4)
None of the above		1005 (42.1)
Not sure/not formally diagnosed		159 (6.7)
Prefer not to say		24 (1)
Knowledge^d^		
Internet search history		2143 (89.7)
Internet browsing history		2141 (89.6)
Health app data		1717 (63.5)
Online banking details (incorrect)		1021 (42.7)
App store data		1490 (62.3)
Content of emails (incorrect)		1050 (43.9)
Purchases made online		1650 (69)
Location history		1831 (76.6)
Internet searches in “incognito” mode (incorrect)		813 (34)
Video streaming history		1720 (72)
Self-reported familiarity with internet use data		
Not familiar		792 (33.1)
Heard about internet use data		850 (35.6)
Some knowledge		512 (21.4)
Extensive knowledge		236 (9.9)

a GCSE: General Certificate of Secondary Education.

b AS: Advanced Subsidiary.

c NVQ: National Vocational Qualification.

d Number and percentage of participants indicating this is part of internet use data.

### Willingness to Share Internet Use Data 

There were no differences in proportions of respondents willing to share internet use data between the 3 medical conditions (for early detection of cancer=77%; heart disease=74%; depression=74%). When asked about different types of data, participants were most willing to share their health app data (73%‐76%, 95% CI 69.8%‐79.1%), whereas participants were less willing to share other types of data, especially regarding purchases made online (52%‐57%, 95% CI 48.7%‐60.3%) and their location history (58%‐59%, 95% CI 54.2%‐62.2%). Details are provided in Table 2. There was variability in what participants understood to be a part of internet use data, with more than 40% of participants incorrectly believing that the content of their emails (1050/2390, 43.9%) and online banking history (1021/2390, 42.7%) would be included (see Table 1).

**Table 2.** Willingness to share internet use data for research on different medical conditions from a cross-sectional survey in 2025 with UK participants recruited through an online survey panel.

Type	Willingness to share internet use data for research on early detection of…^a^									Comparison, *χ*² (df)	*P* value
	Cancer			Heart disease			Depression				
	Total users, n	Users willing to share, n (%)	95% CI	Total users, n	Users willing to share, n (%)	95% CI	Total users, n	Users willing to share, n (%)	95% CI		
Internet use data in general	795	615 (77.4)	74.4‐80.3	798	587 (73.6)	70.5‐76.6	797	592 (74.3)	71.2‐77.3	4.64 (2,2390)	.18
Internet search history	779	532 (68.3)	65.0‐72.6	777	509 (65.5)	62.2‐68.9	787	541 (68.7)	65.5‐72.0	2.18 (2,2343)	.34
Internet browsing history	784	519 (66.2)	62.9‐69.5	783	493 (63.0)	59.6‐66.4	792	535 (67.6)	64.3‐70.8	3.87 (2,2359)	.14
Health app data	682	498 (73.0)	69.7‐76.4	670	508 (75.8)	72.6‐79.1	688	503 (73.1)	69.8‐76.4	1.78 (2,2040)	.41
App store data	730	446 (61.1)	57.6‐64.6	732	455 (62.2)	58.6‐65.7	748	490 (65.5)	62.1‐68.9	3.37 (2,2210)	.19
Purchases made online	770	402 (52.2)	48.7‐55.7	777	414 (53.3)	49.8‐56.8	788	448 (56.9)	53.4‐60.3	3.72 (2,2335)	.16
Location history	771	453 (58.8)	55.3‐62.2	768	443 (57.7)	54.2‐61.2	774	447 (57.8)	54.3‐61.2	0.23 (2,2313)	.89
Video streaming history	750	463 (61.7)	58.3‐65.2	751	471 (62.7)	59.3‐66.2	756	499 (66.0)	62.6‐69.4	3.26 (2,2257)	.20

a Total numbers and percentages for willingness to share include valid participants who are using these services and answered, “definitely yes” and “probably yes.”

Participants were descriptively less willing to share their internet use data if a pictorial example of internet use history was provided alongside written text compared with written text only, but this did not reach statistical significance for research on any of the 3 medical conditions (see Table 3).

Having a personal or family history of the condition being researched was not associated with willingness to share internet use history for that condition, with the exception of those with a personal history of depression. Participants who had a history of depression were more likely to be willing to share their internet use data for the early detection of depression (79.7%, 95% CI 74.5%‐84.8%) than participants without a history of depression (72.2%, 95% CI 67.8%‐76.5%; *P*=.03; Table 3).

**Table 3.** Willingness to share internet use data in relation to how the internet search history was described and personal and family history of the medical condition being researched from a cross-sectional survey in 2025 with UK participants recruited through an online survey panel.

Characteristic		Willingness to share internet use data for research on early detection of…^a^											
		Cancer				Heart disease				Depression			
		N (%)	95% CI	Comparison, *χ*² (df)	*P* value	N (%)	95% CI	Comparison, *χ*² (df)	*P* value	N (%)	95% CI	Comparison, *χ*² (df)	*P* value
Description of internet use				3.56 (1,795)	.06			2.05 (1,798)	.15			2.02 (1,797)	.16
Written description		312 (80.2)	76.2‐84.2			298 (75.8)	71.6‐80.1			294 (76.6)	72.3‐80.8		
Pictorial example and written description		303 (74.6)	76.2‐84.2			289 (71.4)	66.9‐75.8			298 (72.2)	67.8‐76.5		
Personal history of medical condition being researched				3.60 (1,790)	.058			0.36 (1,790)	.55			4.82 (1,794)	.03
Yes		116 (83.5)	77.2‐89.7			103 (75.7)	68.4‐83.0			188 (79.7)	74.5‐84.8		
No		495 (76.0)	72.8‐79.3			479 (73.2)	69.8‐76.6			403 (72.2)	68.5‐76.0		
Family history of medical condition being researched				3.17 (1,792)	.08			0.95 (1,789)	.33			3.17 (1,792)	.08
Yes		171 (81.8)	76.6‐87.1			159 (76.4)	70.6‐82.3			133 (76.9)	70.5‐83.2		
No		442 (75.8)	72.3‐79.3			424 (73.0)	69.4‐76.6			449 (73.4)	69.9‐76.9		

a Total numbers and percentages for willingness to share include participants answering, “definitely yes” and “probably yes.”

### Key Factors Associated With Willingness to Share Internet Use Data

Multivariable logistic regression analysis on willingness to share internet use data for early detection of cancer indicated that willingness to share was associated with perceived benefits and perceived concerns of data sharing. Higher endorsement of benefits of sharing data was associated with a greater willingness to share (OR 8.588, 95% CI 5.687‐12.970). A higher endorsement of concerns was associated with a lower willingness to share (OR 0.432, 95% CI 0.290‐0.643). Ethnicity was significantly associated with willingness to share, with individuals of Asian or Asian British ethnicity demonstrating a significantly lower willingness to share than White participants (OR 0.234, 95% CI 0.076‐0.723). No other demographic factors, including age, gender, employment, income, marital status, education level, personal or family history of cancer, or level of deprivation, were associated with a willingness to share in the multivariable regression analysis, although some (education, employment, ethnicity and income) had indicated associations in univariate analyses (see Multimedia Appendix 1). Factors related to digital literacy (knowledge about internet use data, the number of online services used, or self-reported familiarity with internet use data) were not significantly related to willingness to share internet use data for cancer research in the multivariable regression analysis.

Willingness to share internet use data for the early detection of heart disease and depression followed similar patterns, with perceived benefits being significantly associated with a higher willingness to share internet use data (heart disease: OR 5.692, 95% CI: 4.050‐7.998; depression: OR 8.850, 95% CI 5.998‐13.059) and perceived concerns being significantly associated with a lower willingness to share internet use data (heart disease: OR 0.424, 95% CI 0.292‐0.617; depression: OR 0.343, 95% CI 0.228‐0.516). For research on detection of heart disease, self-reported familiarity with internet use data was associated with a lower willingness to share internet use data (OR 0.740, 95% CI 0.561‐0.976). For research on the early detection of depression, older participants were less willing to share their data (OR 0.975, 95% CI 0.951‐0.999), and men were more likely to be willing to share than women (OR 2.615, 95% CI 1.511‐4.526).

All results are displayed in Table 4.

Sample sizes for the logistic regression analyses were lower than intended due to missing sociodemographic data, especially for levels of deprivation. We therefore conducted sensitivity analyses by repeating the logistic regression analyses without levels of deprivation. Results were similar to the initial logistic regression analysis (see Multimedia Appendix 1), except for depression, where age was no longer associated with willingness to share their data (OR 0.981, 95% CI 0.959‐1.003) and those with a higher knowledge score about internet use data were less willing to share internet use data (OR 0.842, 95% CI 0.723‐0.981). For early detection of cancer, Asian and Asian British were not significantly less likely to share their internet use data (OR 0.351, 95% CI 0.122‐1.011).

**Table 4.** Logistic regression on willingness to share internet use data for research on the early detection of cancer from a cross-sectional survey in 2025 with UK participants recruited through an online survey panel.

Variable	Willingness to share internet use data for research on early detection of…													
	Cancer (n=670)^a^						Heart disease (n=646)^b^				Depression (n=673)^c^			
	Willing, n (%)	OR^d^ (95% CI)	SE	*P* value			Willing, n (%)	OR (95% CI)	SE	*P* value	Willing, n (%)	OR (95% CI)	SE	*P* value
Description of internet use														
Written description	269 (80.5)	1	—^e^	—			246 (78.8)	1	—	—	244 (76.0)	1	—	—
Pictorial example and written description	257 (76.5)	0.617 (0.357-1.066)	0.279	.08			243 (72.8)	0.735 (0.450-1.198)	0.250	.22	263 (74.7)	1.200 (0.723-1.989)	0.258	.48
Gender														
Women	240 (80.5)	1	—	—			228 (74.8)	1	—	—	224 (70.2)	1	—	—
Men	286 (76.9)	0.980 (0.560-1.716)	0.286	.94			261 (76.5)	1.017 (0.607-1.704)	0.263	.95	283 (79.9)	2.615 (1.511-4.526)	0.280	<.001
Education														
Degree level or above	296 (80.9)	1	—	—			263 (79.7)	1	—	—	294 (80.1)	1	—	—
Basic education	98 (72.1)	0.590 (0.300-1.159)	0.344	.13			101 (71.6)	0.688 (0.343-1.380)	0.36	.29	90 (67.7)	0.614 (0.291-1.296)	0.381	.20
Advanced education	132 (78.6)	0.754 (0.390-1.455)	0.336	.40			125 (71.4)	0.627 (0.347-1.130)	0.301	.12	123 (71.1)	0.689 (0.362-1.313)	0.329	.26
Employment														
Employed	324 (83.7)	1	—	—			297 (76.3)	1	—	—	329 (78.5)	1	—	—
Unemployed	69 (67.6)	0.730 (0.353-1.509)	0.371	.40			60 (74.1)	1.172 (0.518-2.656)	0.417	.70	66 (75.0)	1.303 (0.575-2.950)	0.417	.53
Retired	133 (73.5)	1.194 (0.521-2.732)	0.422	.68			132 (75.0)	1.251 (0.583-2.687)	0.390	.57	112 (67.5)	1.770 (0.826-3.792)	0.389	.14
Income (£)														
<35,000	251 (74.5)	1	—	—			254 (73.6)	1	—	—	239 (72.2)	1	—	—
>35,000	275 (82.6)	1.327 (0.703-2.503)	0.324	.38			235 (78.1)	0.826 (0.459-1.487)	0.300	.52	268 (78.4)	0.775 (0.406-1.479)	0.330	.44
Marital status														
Married or in legal partnership	310 (80.9)	1	—	—			299 (79.7)	1	—	—	314 (79.1)	1	—	—
Single	131 (74.4)	1.250 (0.625-2.499)	0.353	.53			123 (70.7)	0.669 (0.364-1.229)	0.310	.20	128 (69.6)	1.020 (0.537-1.936)	0.327	.95
Divorced/ widowed/ separated	85 (76.6)	1.057 (0.511-2.186)	0.371	.88			67 (69.1)	0.754 (0.362-1.571)	0.374	.45	65 (70.7)	1.294 (0.575-2.915)	0.414	.53
Ethnicity														
White	445 (78.1)	1	—	—			416 (75.0)	1	—	—	408 (73.8)	1	—	—
Asian or Asian British	21 (65.6)	0.234 (0.076-0.723)	0.576	.01			22 (81.5)	0.843 (0.231-3.084)	0.661	.80	32 (80.0)	1.277 (0.394-4.133)	0.599	.68
Black or Black British	48 (92.3)	0.806 (0.189-3.431)	0.739	.77			43 (82.7)	0.478 (0.177-1.291)	0.507	.15	58 (89.2)	0.682 (0.225-2.065)	0.565	.50
Mixed or multiple ethnic groups	12 (75.0)	0.251 (0.062-1.019)	0.714	.053			8 (66.7)	0.474 (0.098-2.283)	0.802	.35	9 (60.0)	0.273 (0.063-1.177)	0.745	.08
Level of deprivation														
IMD^f^ Q1 (most deprived)	107 (80.5)	1	—	—			107 (78.7)	1	—	—	120 (78.4)	1	—	—
IMD Q2	130 (77.4)	1.664 (0.766-3.613)	0.396	.20			113 (75.8)	1.116 (0.541-2.302)	0.370	.77	114 (79.7)	2.043 (0.910-4.583)	0.412	.08
IMD Q3	93 (76.9)	0.965 (0.417-2.234)	0.428	.93			99 (72.3)	1.153 (0.531-2.503)	0.395	.72	93 (73.8)	1.450 (0.638-3.295)	0.419	.38
IMD Q4	94 (77.0)	1.162 (0.495-2.729)	0.436	.73			75 (75.0)	1.868 (0.809-4.315)	0.427	.14	83 (69.7)	1.896 (0.811-4.432)	0.433	.14
IMD Q5 (least deprived)	102 (81.0)	1.943 (0.793-4.763)	0.457	.15			95 (76.6)	1.155 (0.512-2.603)	0.415	.73	97 (73.5)	1.142 (0.496-2.628)	0.425	.76
Personal history of condition of interest														
No	422 (77.3)	1	—	—			395 (75.1)	1	—	—	349 (73.0)	1	—	—
Yes	104 (83.9)	1.526 (0.720-3.236)	0.383	.27			94 (78.3)	0.892 (0.444-1.794)	0.357	.75	158 (81.0)	1.345 (0.720-2.513)	0.319	.35
Family history of condition of interest														
No	370 (76.8)	1	—	—			352 (73.9)	1	—	—	390 (74.4)	1	—	—
Yes	156 (83.0)	1.189 (0.651-2.171)	0.307	.57			137 (80.6)	1.037 (0.564-1.907)	0.311	.91	117 (78.5)	1.458 (0.758-2.804)	0.334	.26
Age	—	0.983 (0.958-1.009)	0.013	.21			—	0.984 (0.962-1.008)	0.012	.18	—	0.975 (0.951-0.999)	0.012	.04
Perceived benefits about sharing internet use data	—	8.588 (5.687-12.970)	0.210	<.001			—	5.692 (4.050-7.998)	0.174	<.001	—	8.850 (5.998-13.059)	0.198	<.001
Perceived concerns about sharing internet use data	—	0.432 (0.290-0.643)	0.203	<.001			—	0.424 (0.292-0.617)	0.191	<.001	—	0.343 (0.228-0.516)	0.208	<.001
Knowledge about internet use data (score)	—	1.061 (0.906-1.242)	0.080	.46			—	1.011 (0.866-1.180)	0.079	.891	—	0.858 (0.727-1.012)	0.084	.07
Number of online services used	—	0.923 (0.826-1.031)	0.057	.16			—	1.040 (0.942-1.148)	0.051	.440	—	1.033 (0.929-1.149)	0.054	.55
Familiarity with internet use data	—	1.108 (0.814-1.509)	0.158	.52			—	0.740 (0.561-0.976)	0.141	.033	—	0.854 (0.625-1.167)	0.160	.32

a Nagelkerke *R=*0.573.

b Nagelkerke *R=*0.516

c Nagelkerke *R=*0.588.

d OR: odds ratio.

e Not applicable.

f IMD: index of multiple deprivation.

### Statements on Willingness to Share and Concerns

**Figure 1.**
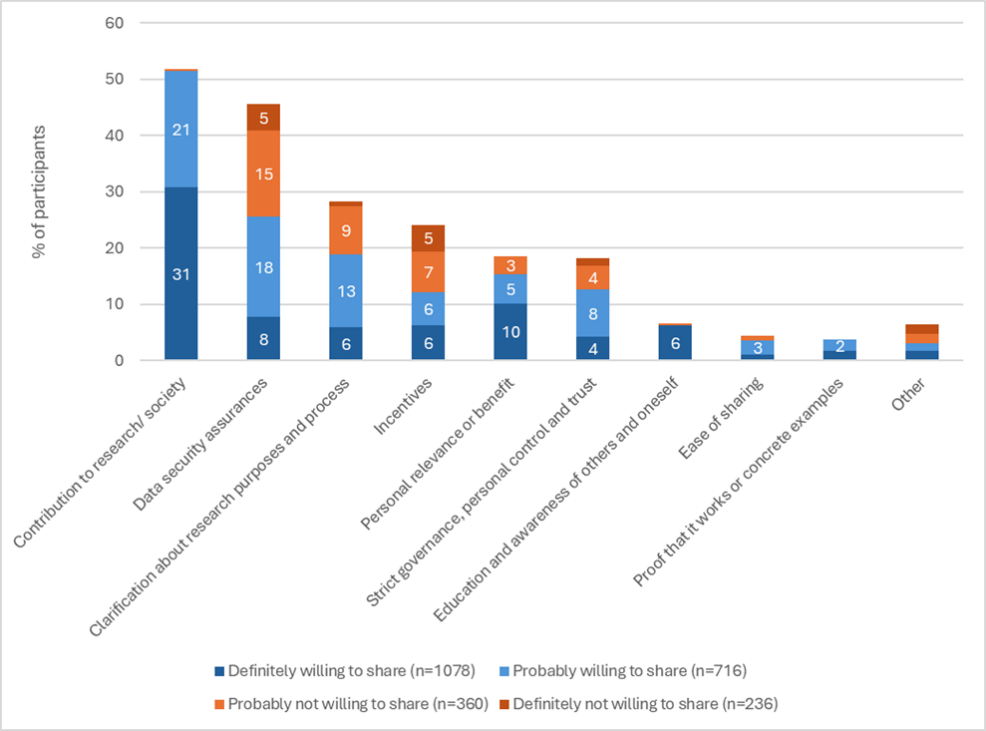
Suggestions to increase willingness to share internet use data from a cross-sectional survey in 2025 with UK participants recruited through an online survey panel. Statements could be coded as multiple categories. Participants saying nothing would make them more willing are not included in this figure.

The open-ended question on what would make them more willing to share their internet use data was answered by 2307 (2307/2390, 96.5%) participants. Percentages of statements relative to numbers of those definitely willing, probably willing, probably unwilling, and definitely unwilling to share are presented in Figure 1. The main suggestions given by those currently not willing to share were strong assurance of data security (66/596, 11.1%) and incentives (37/596, 6.2%); however, the majority of these participants stated “nothing” would make them more willing (definitely not: 189/236, 80.1%; probably not: 196/360, 54.4%). Those who were “probably” or “definitely” willing to share their internet use data offered a wider range of suggestions, including a sense of contribution to research and society (“helping others”: 480/1794, 26.8%); data security assurances (211/1794, 11.8%); clarification of research purposes (157/1794, 8.6%); incentives (110/1794, 6.1%); strict governance, personal control, and building trust (106/1794, 5.9%); and personal relevance (147/1794, 8.2%).

**Figure 2.**
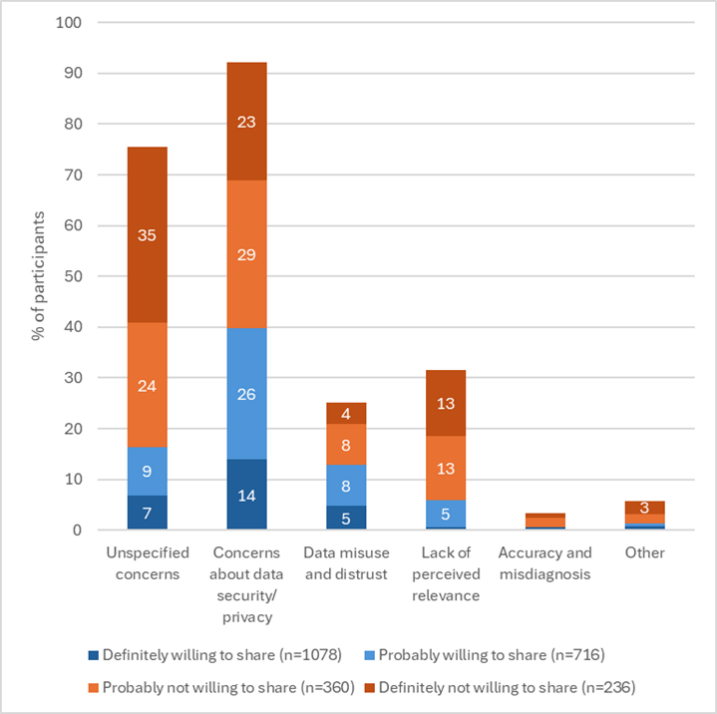
Concerns about sharing internet use data from a cross-sectional survey in 2025 with UK participants recruited through an online survey panel. Statements could be coded as multiple categories. Participants saying they had no concerns are not included in this figure.

The question about concerns surrounding sharing internet use data was answered by 2318 (2318/2390, 97%) participants (Figure 2). Concerns of participants who were “probably not” or “definitely not” willing to share their internet use data mostly focused on data security and the invasion of privacy (160/596, 26.8%) and concerns about data misuse, including sharing with private (marketing) companies (39/596, 6.5%). Participants also questioned the relevance of their internet use data for early detection of disease (76/596, 12.8%). Participants who were “probably” or “definitely” willing to share their internet use data noted similar concerns, although many indicated no concerns (probably willing: 368/716, 51.4%; definitely willing: 794/1078, 73.7%). Examples are provided in Multimedia Appendix 1.

## Discussion

### Principal Findings

This study examined willingness to share internet use data for research into early detection of cancer, heart disease, and depression. Participants showed a similarly high willingness to share internet use data for all conditions. Willingness differed by data type, being lowest for online purchases and location history and highest for health app data. Viewing a screenshot of browsing history did not significantly affect willingness for any condition.

Perceived benefits and concerns were key factors associated with willingness across all conditions. Answering open questions about willingness to share, participants reported that contributing to research, personal relevance, data security, incentives, clear research purposes, concrete examples, strong governance, and trust in researchers would improve their willingness to share. In contrast, they reported that privacy concerns, fear of misuse, and low relevance could reduce willingness to share. Those who were more familiar with internet use data were less likely to share data for heart disease detection, but this was not associated with willingness to share data for research on cancer or depression. There were few sociodemographic factors associated with willingness to share, but some did exist: Asian and Asian British participants were less willing to share their internet use data for cancer detection (not replicated in the sensitivity analyses), while younger (not replicated in the sensitivity analyses) and male participants were more willing to share data for depression detection.

### Comparison With Prior Work

We found a surprisingly high willingness to share internet use data for health research compared with recent studies. For example, Grande et al [20] classified approximately 13% of their participants as “agreeable to sharing,” whereas about 45% of our participants indicated they were “definitely” willing to share. Similarly, Hirst et al [19] found that 61% of participants were willing to share their data with research organizations, and only 43% were willing to share with private organizations, compared with 75% in this study. Although we did not differentiate the kind of organization data would be shared with, we consistently found that a higher proportion of participants were willing to share across different types of data than in these studies. Our proportions are based on users of the respective types of data. However, willingness to share was similar when participants were asked more generally about “internet use data.”

As we used a similar recruitment strategy as previous studies [19], it seems that the *purpose* of the data sharing described in our study may be responsible for the higher willingness to share internet use data. In our survey, we asked participants specifically about sharing their internet use data for the early detection of health conditions. Previous research has found public support for screening and early detection [35,36], especially if personally relevant [37]. This is line with open-ended comments regarding increased willingness to share if there were personal benefits (eg, receiving a quicker diagnosis). Beyond personal benefits, participants in our study who were willing to share their internet use data described a general sense of contribution to research and society, which our PPI group summarized as an act of “charity” in terms of “donating data.” The Smart Data Research UK report [12] reports how the public grew more enthusiastic about using smart data in research for the “public good” when learning and discussing potential applications. However, information about the value of data sharing, as well as ensuring personal control over sharing potentially sensitive information is crucial to ensure acceptability of sharing data [38], which is reflected in our findings.

Another suggestion for improving willingness to share internet use data in our study was monetary incentives, which was not captured in our quantitative scales but reported in the free text. Even participants who were currently unwilling to share mentioned incentives as potentially increasing their willingness to share. This indicates a “trade-off” between privacy and payment, although the exact value of individual privacy is difficult to determine [39].

Our findings regarding higher willingness to share health app data than to share other types of internet use data are in line with previous research [20]. For example, Ackermann et al [40] found that a better “intuitive match” between the type of data requested and what the data are used for increases willingness to share internet use data. In this survey study, this would be the case for health app data but not necessarily for online purchases or location data, which participants were less willing to share. However, researchers developing risk prediction models based on or including internet use data might rely on changes in online behavior beyond concrete health app data (eg, if an individual repeatedly searches for nonspecific symptoms or home remedies). As individuals are likely to look up symptoms online or change the tone and semantics of their searches long before visiting their general practitioner, capturing these early changes might benefit early detection [13-16].

Types of data that are not directly related to medical contexts might also be seen as more personal and more likely to be misused, such as by marketing companies [19]. Concerns about privacy and misuse of data were associated with a lower willingness to share across all conditions in this study. They were commonly endorsed and reported in both the quantitative and qualitative part of the survey, even by participants who were “definitely” willing to share. The Smart Data Research UK report [12] also shows that the public is skeptical about private sector motivation and concerned about potential inequalities and data security.

### Implications for Future Research and Practice

There is a clear need for transparent and open communication around the purpose of using internet data for early detection research, as well as data processing after it is shared, including which data, how, and why they are collected and stored [12,38]. Researchers and data scientists need to work toward clarifying which data are needed for what purpose, how to limit data sharing to what is necessary and filter out any other information, and how to communicate purpose and process of data sharing, especially concerning minority groups [41,42]. We found no effect of showing participants a pictorial example of internet use data on willingness to share. Our PPI group noted that the example only included a generic internet history without particularly sensitive data (eg, regarding financial details or a child’s local school). Future research could examine the effect of different types of examples, preferably using participants’ own internet use data.

Only 10% of participants in our sample noted that they knew a lot about internet use data, and 34% to 44% mistakenly believed that internet searches in “incognito” mode, online banking details, and content of emails are part of internet use data. This indicates that internet users might not be well-informed about the personal data they might be sharing online [43,44]. There is a need for more public information and education around what internet use data entail and what would need to be accessed for medical research to facilitate informed consent [45]. Our findings suggest that higher familiarity and, potentially, knowledge might negatively impact willingness to share internet use data for medical research. However, this should be interpreted with caution, as univariate analyses indicated the opposite trend. Actual familiarity and knowledge about internet use data and the consequences for willingness to share data should be investigated in more detail. Clear communication including which data are shared with whom and ensuring personal control are central to enable informed participation in internet research [45].

Our findings show how concerns around sharing internet use data might be more pronounced in some ethnic minority groups and corroborate research showing that cancer is associated with fear and stigma in minority communities [46]. Future research should focus on mitigation strategies to avoid inequalities in this new stream of internet-based medical research, especially for vulnerable populations. Stigma around mental health conditions might drive the findings that younger age and male gender were associated with a higher willingness to share internet use data for the detection of depression [47]. As traditional norms of masculinity can reduce symptom expression and inhibit help-seeking for mental health conditions [48,49], sharing internet use data might be a more anonymous way to disclose potential symptoms.

Although many participants reported having concerns about sharing internet use data, a high proportion did not specify their concerns. This might have been enforced by the wording of the open-ended question around concerns but could also reflect a general unease with data sharing due to a lack of knowledge and transparency [50]. A qualitative study identified similar concerns around data security and misuse [51] as well as actions taken by individuals to protect their privacy, such as use of incognito mode for sensitive searches, that could impact future research. Future research, especially qualitative research, should examine how these concerns can be mitigated in more detail. Additionally, those who reported being “definitely not” willing to share data often said “nothing” would make them more willing. Future research should therefore investigate why some people would not be willing to share internet use data under any circumstances.

### Strengths and Limitations

This study is a large survey with population-based quotas that builds on a previous qualitative study [51], previously used measures [18,19,24], and extensive patient and public input. However, the survey assessed self-reported willingness to share rather than the actual behavior, as well as self-reported diagnosis, which might have led to an overestimation of those with a confirmed clinical diagnosis, especially in those reporting to have depression [52]. It remains to be seen whether these results will be confirmed in future research and practice when shared internet use data are required.

We recruited through an online panel and therefore included participants who are comfortable answering online research surveys for incentives and attracted those with higher education (approximately 52% with at least a degree level of education, compared with the UK average of 33%‐34%, [53]). This might have contributed to the high willingness to share internet use data and frequent mentioning of incentives to increase willingness in the open-ended questions. Nevertheless, internet use data can only be shared by people who are using the internet in the first place, and internet use and knowledge about internet use data varied in the sample, suggesting the sample represents a range of internet users. The power for some of the logistic regression analyses might have been too low to detect differences. Although we ensured a representative percentage of the sample (approximately 17%) was from ethnic minority groups, the numbers within minority ethnic groups limit accurate comparisons.

Other potential methodological limitations due to the short survey design include the use of area-based index of multiple deprivation quintiles for individual-level data, potentially leading to ecological fallacy as individuals living in affluent areas may still experience deprivation and vice versa [54]. Nevertheless, it is still the most comprehensible method that could be applied in a short questionnaire, and sensitivity analyses were used to replicate logistic regression analyses without this deprivation variable [55]. We combined those “definitely” and “probably” (not) willing to share for analyses, although the groups might differ. Dichotomizing questions about willingness to share internet use data might have reduced the nuance of the data.

Finally, we had to exclude a substantial proportion (16.6%) of responses due to low data quality, including those flagged as bots and fraudulent responses and those failing attention checks. This does not negatively impact the quality of our final sample, as we were able to exclude and replace these participants during recruitment. However, this shows that researchers will need to account for “impostor” responses when planning for sample sizes for online surveys, implement strategies to detect low quality data (eg, attention checks or bot detection), and carefully check responses, leading to additional workload. If low-quality responses are not addressed, we may not be able to trust similar research results [56].

### Conclusion

This study is innovative as the first large-scale survey, to the best of our knowledge, to examine public willingness to share internet use data for early detection research across multiple common health conditions while assessing perceived benefits, risks, and sociodemographic differences. This survey indicated willingness to share internet use data is high across research on early detection of different common health conditions. Willingness to share was associated with perceived benefits for oneself and society in general and limited by concerns around data privacy and potential for misuse. Participants were more willing to share data that were plausibly related to the purpose of early detection of health conditions (eg, health app data).

The survey results advance the understanding of participation in research using internet use data by identifying both barriers and enablers of data sharing. Although sociodemographic differences were limited, variations by age, gender, and ethnicity suggest potential inequities. The findings inform consent design, communication strategies, and data governance, highlighting the need for clear purpose limitation and robust data protection to foster trust and reduce inequalities. They emphasize the need to clarify which and how data are used for research into early detection and investigate how communication about internet use data could improve willingness to share while avoiding perpetuating inequalities. Data sharing needs to be limited to specific research purposes, and resolute data protection strategies need to be put in place.

## Supplementary material

10.2196/85637 Multimedia Appendix 1 WISER survey, additional regression analyses, and definitions of categories for suggestions to increase willingness to share and for concerns.

## Abbreviations

GDPR: General Data Protection Regulation
JARS-Quant: Journal Article Reporting Standards Quantitative Research Design
OR: odds ratio
OSF: Open Science Framework
PPI: patient and public involvement
