# Supplementary material for: Willingness to Share Internet Use Data for Research on Early Disease Detection: Cross-Sectional Survey
Source: J Med Internet Res. 2026 Mar 25;28:e85637. doi: 10.2196/85637 (PMC13016547; doi:10.2196/85637)
Supplement: Multimedia Appendix 1 [file jmir-v28-e85637-s001.docx]

# WISER survey

## Introduction

Thank you for your interest in this research.

The aim of this study is to find out how the public feel about health research that looks at people’s internet use to help detect early signs of serious health conditions such as cancer, heart disease or depression.

For this study, we will NOT ask you to share any of your internet use data with us and does NOT commit you to sharing data at any time in the future. We only want to find out what kind of research you might be willing to share your data for, and your views on this type of research.

Your answers will be anonymous – we will not be able to identify you.

The survey will take 15 to 20 minutes. You can find more information, including how we save and process your answers, under this link: PIS.

If you have any questions or concerns, please contact the researcher Christina Derksen ([christina.derksen@qmul.ac.uk](mailto:christina.derksen@qmul.ac.uk), XXXXX XXXXXX).


Should you wish to participate in the study, please consider the following statements. 

[CONSENT]

## Random assignment to one of 6 (3x2) survey conditions

1. Cancer – *concrete example* of internet use data
2. Cancer – *no example* of internet use data
3. Heart disease - *concrete example* of internet use data
4. Heart disease – *no example* of internet use data
5. Depression – *concrete example* of internet use data
6. Depression – *no example* of internet use data

**No example**

Internet use data includes information on how you engage with the internet. Researchers are interested to find out if internet use data could help detect early signs of serious health conditions such as cancer, heart disease or depression.

**Concrete example**

Internet use data includes information on how you engage with the internet - what you search for, what you click on, when you engage online, what internet sites you visit and what you download. Researchers are interested to find out if internet use data could help detect early signs of serious health conditions such as cancer, heart disease or depression.

Please have a look at this example of internet search data that includes Google searches, visited websites, and videos a person watched:


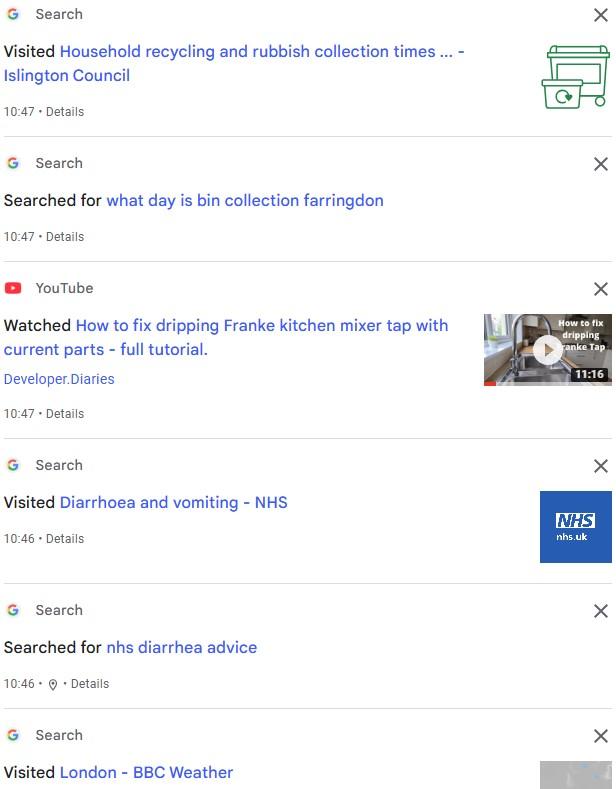


## Survey

| **Construct** | **Items** | **Answer options** | **Comments** |
| --- | --- | --- | --- |
| Willingness to share internet use data | Would you be willing to share your internet use data for research on early diagnosis of [randomly assigned condition]? | - Definitely Yes  - Probably Yes  - Probably No  - Definitely No |  |
| Improving willingness to share | What would make you more willing to share your internet use data for research on early diagnosis of [ randomly assigned condition]? | Open-ended |  |
| Concerns about sharing data | Would you have any concerns about sharing your internet use data for research on early diagnosis of [ randomly assigned condition]? | Open-ended | Suggested by PPI representatives |
| Attitudes towards sharing data (benefits) | To what extent do you agree with the following statements?  1) Sharing my data could help future generations.  2) Sharing my data could lead to better future medical treatments.  3) Sharing my data could improve the patients' health outcomes.  4) Sharing my data could help my family and friends.  5) Sharing my data could help me personally. | - Strongly agree  - Agree  - Neither agree nor disagree  - Disagree  - Strongly disagree | Taken from Sanderson et al^1^ |
| Attitudes towards sharing data (concerns) | To what extent do you agree with the following statements?  1) I would worry that my data could not be anonymised easily.  2) I would worry about not being able to control the timeframe for which I share my data (e.g., restricting shared data to last few months).  3) I would worry that my data would not be relevant enough to use it for research.  4) I would worry that data that is relevant for research could not be easily separated from other data.  5) I would worry that my data would not be protected from those who should not have access to it.  6) I would worry researchers might use my data for purposes not stated in the consent or privacy policy.  7) I would worry my data will be shared with others without my consent.  8) I would worry someone would use my data to make money.  9) I would fear that someone could use my data against me.  10) I would be embarrassed to share my data.  11) I would not know how to share my data. | - Strongly agree  - Agree  - Neither agree nor disagree  - Disagree  - Strongly disagree | Taken from Hirst et al^2^, who adapted from Sanderson et al^1^ and Seltzer et al^3^ |
| Awareness of internet use data: Heard of it? | Have you heard of internet use data before taking part in this survey? | - No, I was not aware.  - Yes, I have heard about it but do not know much.  - Yes, I have heard about it, and I know a little.  - Yes, I have heard about it, and I know a lot. | Adapted from Hirst et al^2^ |
| Attention check | Please select "strongly agree" to show that you are paying attention. | - Disagree  - Strongly agree  - Strongly disagree  - Neither agree nor disagree  - Agree |  |
| Awareness of internet use data: Knowledge | Which of the following do you think are part of your internet use data?   1. Internet search history (what you searched for) 2. Internet browsing history (websites you looked at) 3. Health app data (e.g., Fitbit data) 4. Online banking details 5. App store data 6. Content of emails 7. Purchases made online 8. Location history such as Google Maps 9. Internet searches in “incognito” mode 10. Video streaming history (e.g. what you have watched on YouTube) | - Yes  - No  - I don’t know |  |
| Willingness to share different types of internet use data | Would you be willing to share the following parts of your internet use data for research on early diagnosis of [ randomly assigned condition]?   1. Internet search history (what you searched for) 2. Internet browsing history (websites you looked at) 3. Health app data (e.g., Fitbit data) 4. App store data 5. Purchases made online 6. Location history such as Google Maps 7. Video streaming history (e.g. what you have watched on YouTube) | - Definitely Yes  - Probably Yes  - Probably No  - Definitely No  - Not applicable (I do not use this) |  |
| Internet use | How often do you access the internet? | - Regularly  - Occasionally  - Rarely  - Never | <https://www.gov.uk/government/publications/digital-landscape-research/digital-landscape-research#annex-1-data> |
| Use of online services | Which of the following services do you regularly use online? | - Email/messenger  - Search engines  - Online shopping  - News and weather  - Online banking  - Social networking  - Price comparison  - General information  - Government information and services  - Entertainment/ Gaming  - Travel booking sites  - Other [option to specify] | <https://www.gov.uk/government/publications/digital-landscape-research/digital-landscape-research#annex-1-data> |
| Honeypot question for bot detection | Please write your age in years. | Open-ended |  |
| We would like to ask you some questions so we can understand who is taking part in our research. We would like to understand if some people are more willing to share their data than others. Please respond to as many questions as you feel comfortable. Your answers will remain confidential and will not affect you or your participation in this project in any way. If you would rather not answer any question, please select “prefer not to say”. | | | |
| Socio-demographic questions: Age | How old are you (in years)? | Open box |  |
| Socio-demographic questions: Gender | Which of the following best describes your gender? | - Man  - Woman  - Non-binary  - Prefer not to say  - Prefer to self-describe [option to specify] | Taken from DISTINCT set of items^4^ |
| Socio-demographic questions: Educational level | Have you completed any qualifications?  If you did not complete your education in the UK, please choose the closest equivalent. This is not a complete list. Please choose the options that best fit you. | - No formal qualifications  - GCSEs or equivalent (e.g. GCSEs, O levels, CSEs, Basic Skills course)  - AS, A level or equivalent  - NVQ up to level 3 or equivalent (e.g. NVQ level 1-3, BTEC General or National, OND or ONC, City and Guilds Advanced Craft)  - Qualification at degree level or above (e.g. university degree, foundation degree, HND, HNC, NVQ level 4 or above, teaching or nursing)  - Apprenticeship (e.g. trade, advanced, foundation, modern)  - Any other qualifications or equivalent unknown  - Prefer not to say | Taken from DISTINCT set of items^4^ |
| Socio-demographic questions: Employment | Please select all that apply: | - Employed (including self-employed)  - Not employed  - Retired  - On temporary leave (e.g., parental leave)  - Studying  - Prefer not to say | Taken from DISTINCT set of items^4^ |
| Socio-demographic questions: Income | What is your total disposable household income per year, including all earners in your household (after tax) in GBP? | - Under £35,000  - Over £35,000  - Prefer not to say |  |
| Socio-demographic questions: marital status | What is your current marital/ registered civil partnership status? | - Single  - Married or in legal partnership  - Widowed, divorced, separated  - Prefer not to say | Hirst et al.^2^ |
| Socio-demographic questions: ethnicity | What is your ethnic group?  Please choose one option that best describes your ethnic group or background. | - Asian or Asian British  - Black, Black British, Caribbean or African  - Mixed or multiple ethnic groups  - White  - Other [option to specify]  - Prefer not to say | Taken from DISTINCT set of items^4^ |
| Socio-demographic questions: preferred language | What is your preferred language? | - English  - Other [option to specify]  - Prefer not to say | Taken from DISTINCT set of items^4^ |
| Socio-demographic questions: Postcode | Please indicate your postcode. We will use it to group together the answers for everyone living in particular areas. | Open-ended |  |
| History of cancer, CVD and mental health issues | Do you have a history of any of the following: | - Cancer  - Heart disease or vascular problems (e.g. hypertension, thrombosis, stroke)  - Depression  - I am not sure/ not formally diagnosed  - Prefer not to say | Answer options adapted from:  <https://digital.nhs.uk/data-and-information/publications/statistical/health-survey-for-england/2021-part-2/adult-health-general-health> |
| Experiences of friends and family members with cancer, CVD and mental health issues | Do any of *your* *relatives or close friends* have any of the following: | - Cancer  - Heart disease or vascular problems (e.g. hypertension, thrombosis, stroke)  - Depression  - I don’t know  - Prefer not to say | Answer options adapted from:  <https://digital.nhs.uk/data-and-information/publications/statistical/health-survey-for-england/2021-part-2/adult-health-general-health> |

## Exit page

 Thank you for participating in our survey!

This is all part of your internet use data:

1) Internet search history (what you searched for)
2) Internet browsing history (websites you looked at)
3) Health app data (e.g., Fitbit data)
4) App store data
5) Purchases made online
6) Location history such as Google Maps
7) Video streaming history (e.g. what you have watched on YouTube)

This is not part of your internet use data:
1) Online banking details (other than which app/ website you used)
2) Content of emails
3) Internet searches in “incognito” mode

A report with study findings will published through our Cancer Prevention and Screening Blog in a few months.

**If you have any queries or concerns, please contact the researcher Christina Derksen (**[**christina.derksen@qmul.ac.uk**](mailto:christina.derksen@qmul.ac.uk)**, XXXXX XXXXXX).**

Future research might ask people to share their internet use data, especially internet search history, to find out if could be used to help to detect early signs of serious health conditions. For example, someone may a patient could google look up symptoms or buy over-the-counter medication online a long time before going to the GP. By analysing internet use data, we researchers might be able to find when it would be helpful to alert help people and their families to visit a doctor earlier. Shared internet use data could be filtered to only include health-related information and would be anonymised before being analysed by researchers.


You can find digital support and more information here:

**AbilityNet**
Online resources and community-based volunteers who help older individuals and individuals with a disability of any age to use technology. You can reach them:
• By phone: 0300 180 0028 during UK office hours
• By email: enquiries@abilitynet.org.uk
• Or request help online: https://abilitynet.org.uk/free-tech-support-and-info/request-free-it-support-home

**Good Things Foundation**
Online resources to help gain basic digital skills.
• Learn My Way resources: https://www.learnmyway.com/
• Find local support near you: https://www.goodthingsfoundation.org/

**Age UK**
Have a number of resources to help you get started, make the most of what is available, and stay safe online.
• https://www.ageuk.org.uk/information-advice/work-learning/technology-internet/

**National Cyber Security Centre**
Provide advice and guidance on staying secure online.
• https://www.ncsc.gov.uk/collection/top-tips-for-staying-secure-online

# References used in this document

1. Sanderson SC, Brothers KB, Mercaldo ND, et al. Public attitudes toward consent and data sharing in biobank research: a large multi-site experimental survey in the US. *The American Journal of Human Genetics*. 2017;100(3):414-427.

2. Hirst Y, Stoffel ST, Brewer HR, Timotijevic L, Raats MM, Flanagan JM. Understanding Public Attitudes and Willingness to Share Commercial Data for Health Research: Survey Study in the United Kingdom. *JMIR Public Health Surveill*. Mar 23 2023;9:e40814. doi:10.2196/40814

3. Seltzer E, Goldshear J, Guntuku SC, et al. Patients’ willingness to share digital health and non-health data for research: a cross-sectional study. *BMC Medical Informatics and Decision Making*. 2019/08/08 2019;19(1):157. doi:10.1186/s12911-019-0886-9

4. The Institute of Cancer Research. DISTINCT: collecting data to monitor inclusivity of clinical research. 21 August, 2025, . <https://www.icr.ac.uk/research-and-discoveries/centres-and-strategic-collaborations/clinical-trials-and-statistics-unit-icr-ctsu/our-research-at-icr-ctsu/clinical-trials-methodology-research/distinct-project>

# Univariate regression analyses

| **Variable** | | **Willingness to share internet use data for research on early detection of…** | | | | | | | | | | | | | | | | | |
| --- | --- | --- | --- | --- | --- | --- | --- | --- | --- | --- | --- | --- | --- | --- | --- | --- | --- | --- | --- |
|  |  | **Cancer** | | | | | | **Heart disease** | | | | | | **Depression** | | | | | |
|  |  | **Willing**  **n (%)** | **OR** | **S.E.** | ***P* value** | **95% C.I. for Odds ratio** | | **Willing**  **n (%)** | **OR** | **S.E.** | ***P* value** | **95% C.I. for Odds ratio** | | **Willing**  **n (%)** | **OR** | **S.E.** | ***P* value** | **95% C.I. for Odds ratio** | |
|  |  |  |  |  |  | **Lower** | **Upper** |  |  |  |  | **Lower** | **Upper** |  |  |  |  | **Lower** | **Upper** |
| Description of internet use | Written description | 269 (80.5) | 1 |  |  |  |  | 246 (78.8) | 1 |  |  |  |  | 244 (76.0) | 1 |  |  |  |  |
|  | Pictorial example and written description | 257 (76.5) | 0.726 | 0.171 | .061 | 0.519 | 1.015 | 243 (72.8) | 0.794 | 0.161 | .153 | 0.579 | 1.089 | 263 (74.7) | 0.793 | 0.163 | .155 | 0.576 | 1.092 |
| Gender | Women | 240 (80.5) | 1 |  |  |  |  | 228 (74.8) | 1 |  |  |  |  | 224 (70.2) | 1 |  |  |  |  |
|  | Men | 286 (76.9) | 0.909 | 0.171 | .575 | 0.651 | 1.270 | 261 (76.5) | 1.214 | 0.162 | .231 | 0.884 | 1.667 | 283 (79.9) | 1.596 | 0.164 | .004 | 1.158 | 2.199 |
| Education | Degree level or above | 296 (80.9) | 1 |  |  |  |  | 263 (79.7) | 1 |  |  |  |  | 294 (80.1) | 1 |  |  |  |  |
|  | Basic education | 98 (72.1) | 0.522 | 0.208 | .002 | 0.347 | 0.786 | 101 (71.6) | 0.631 | 0.209 | .027 | 0.419 | 0.950 | 90 (67.7) | 0.510 | 0.206 | .001 | 0.341 | 0.765 |
|  | Advanced education | 132 (78.6) | 0.825 | 0.211 | .362 | 0.545 | 1.248 | 125 (71.4) | 0.622 | 0.189 | .012 | 0.429 | 0.901 | 123 (71.1) | 0.637 | 0.198 | .023 | 0.432 | 0.939 |
| Employ-ment | Employed | 324 (83.7) | 1 |  |  |  |  | 297 (76.3) | 1 |  |  |  |  | 329 (78.5) | 1 |  |  |  |  |
|  | Unemployed | 69 (67.6) | 0.409 | 0.223 | <.001 | 0.264 | 0.633 | 60 (74.1) | 0.770 | 0.231 | .258 | 0.490 | 1.210 | 66 (75.0) | 0.679 | 0.236 | .101 | 0.427 | 1.079 |
|  | Retired | 133 (73.5) | 0.600 | 0.201 | .011 | 0.405 | 0.890 | 132 (75.0) | 0.862 | 0.190 | .435 | 0.593 | 1.252 | 112 (67.5) | 0.573 | 0.190 | .003 | 0.395 | 0.832 |
| Income | <£35,000 | 251 (74.5) | 1 |  |  |  |  | 254 (73.6) | 1 |  |  |  |  | 239 (72.2) | 1 |  |  |  |  |
|  | >£35,000 | 275 (82.6) | 1.936 | 0.178 | .001 | 1.366 | 2.744 | 235 (78.1) | 1.476 | 0.167 | .020 | 1.063 | 2.048 | 268 (78.4) | 1.531 | 0.166 | .010 | 1.105 | 2.120 |
| Marital status | Married or in legal partnership | 310 (80.9) | 1 |  |  |  |  | 299 (79.7) | 1 |  |  |  |  | 314 (79.1) | 1 |  |  |  |  |
|  | Single | 131 (74.4) | 0.598 | 0.192 | .007 | 0.410 | 0.871 | 123 (70.7) | 0.518 | 0.182 | <.001 | 0.363 | 0.740 | 128 (69.6) | 0.529 | 0.183 | <.001 | 0.370 | 0.757 |
|  | Divorced/ widowed/ separated | 85 (76.6) | 0.736 | 0.242 | .206 | 0.458 | 1.183 | 67 (69.1) | 0.561 | 0.231 | .012 | 0.357 | 0.882 | 65 (70.7) | 0.684 | 0.240 | .114 | 0.427 | 1.095 |
| Ethnicity | White | 445 (78.1) | 1 |  |  |  |  | 416 (75.0) | 1 |  |  |  |  | 408 (73.8) | 1 |  |  |  |  |
|  | Asian or Asian British | 21 (65.6) | 0.650 | 0.361 | .233 | 0.321 | 1.319 | 22 (81.5) | 0.942 | 0.366 | .871 | 0.460 | 1.932 | 32 (80.0) | 1.311 | 0.353 | .443 | 0.656 | 2.617 |
|  | Black or Black British | 48 (92.3) | 2.214 | 0.388 | .040 | 1.035 | 4.734 | 43 (82.7) | 2.184 | 0.353 | .027 | 1.094 | 4.361 | 58 (89.2) | 2.588 | 0.350 | .007 | 1.304 | 5.134 |
|  | Mixed or multiple ethnic groups | 12 (75.0) | 1.201 | 0.567 | .747 | 0.396 | 3.645 | 8 (66.7) | 0.555 | 0.534 | .271 | 0.195 | 1.582 | 9 (60.0) | 0.475 | 0.512 | .146 | 0.174 | 1.296 |
| Level of deprivation | IMD Q1 (most deprived) | 107 (80.5) | 1 |  |  |  |  | 107 (78.7) | 1 |  |  |  |  | 120 (78.4) | 1 |  |  |  |  |
|  | IMD Q2 | 130 (77.4) | 1.089 | 0.261 | .745 | 0.652 | 1.817 | 113 (75.8) | 0.850 | 0.263 | .536 | 0.507 | 1.424 | 114 (79.7) | 0.988 | 0.265 | .965 | 0.587 | 1.663 |
|  | IMD Q3 | 93 (76.9) | 1.048 | 0.282 | .869 | 0.603 | 1.821 | 99 (72.3) | 0.668 | 0.263 | .125 | 0.399 | 1.119 | 93 (73.8) | 0.842 | 0.272 | .528 | 0.494 | 1.435 |
|  | IMD Q4 | 94 (77.0) | 1.035 | 0.280 | .903 | 0.598 | 1.791 | 75 (75.0) | 0.724 | 0.282 | .252 | 0.416 | 1.259 | 83 (69.7) | 0.712 | 0.271 | .211 | 0.419 | 1.212 |
|  | IMD Q5 (least deprived) | 102 (81.0) | 1.250 | 0.290 | .442 | 0.708 | 2.205 | 95 (76.6) | 0.811 | 0.276 | .447 | 0.472 | 1.393 | 97 (73.5) | 0.774 | 0.269 | .341 | 0.456 | 1.312 |
| Personal history of condition of interest | No | 422 (77.3) | 1 |  |  |  |  | 395 (75.1) | 1 |  |  |  |  | 349 (73.0) | 1 |  |  |  |  |
|  | Yes | 104 (83.9) | 1.589 | 0.246 | .069 | 0.981 | 2.574 | 94 (78.3) | 1.140 | 0.219 | .548 | 0.743 | 1.750 | 158 (81.0) | 1.506 | 0.187 | .029 | 1.044 | 2.175 |
| Family history of condition of interest | No | 370 (76.8) | 1 |  |  |  |  | 352 (73.9) | 1 |  |  |  |  | 390 (74.4) | 1 |  |  |  |  |
|  | Yes | 156 (83.0) | 1.436 | 0.204 | .076 | 0.963 | 2.140 | 137 (80.6) | 1.202 | 0.188 | .329 | 0.831 | 1.738 | 117 (78.5) | 1.207 | 0.202 | .352 | 0.812 | 1.794 |
| Age | |  | 0.995 | 0.005 | .317 | 0.985 | 1.005 |  | 0.995 | 0.005 | 0.286 | 0.985 | 1.004 |  | 0.982 | 0.005 | <.001 | .973 | .992 |
| Perceived benefits about sharing internet use data | |  | 8.290 | 0.170 | <.001 | 5.940 | 11.570 |  | 6.515 | 0.142 | <.001 | 4.930 | 8.610 |  | 7.531 | 0.154 | <.001 | 5.569 | 10.185 |
| Perceived concerns about sharing internet use data | |  | 0.407 | 0.120 | <.001 | 0.322 | 0.515 |  | 0.377 | 0.117 | <.001 | 0.300 | 0.473 |  | 0.444 | 0.112 | <.001 | 0.356 | 0.553 |
| Knowledge about internet use data (score) | |  | 1.047 | 0.052 | .372 | 0.946 | 1.159 |  | 1.072 | 0.050 | .158 | 0.973 | 1.182 |  | 0.924 | 0.052 | .127 | 0.835 | 1.023 |
| Number of online services used | |  | 1.058 | 0.032 | .080 | 0.993 | 1.127 |  | 1.061 | 0.030 | .045 | 1.001 | 1.125 |  | 1.013 | 0.031 | .678 | 0.953 | 1.076 |
| Familiarity with internet use data | |  | 1.240 | 0.093 | .020 | 1.034 | 1.487 |  | 1.122 | 0.083 | .167 | 0.954 | 1.321 |  | 1.287 | 0.088 | .004 | 1.083 | 1.528 |

# Regression analyses without levels of deprivation (sensitivity analyses)

| **Variable** | | **Willingness to share internet use data for research on early detection of…** | | | | | | | | | | | | | | | | | |
| --- | --- | --- | --- | --- | --- | --- | --- | --- | --- | --- | --- | --- | --- | --- | --- | --- | --- | --- | --- |
|  |  | **Cancer** | | | | | | **Heart disease** | | | | | | **Depression** | | | | | |
|  |  | **Willing**  **n (%)** | **OR** | **S.E.** | ***P* value** | **95% C.I. for Odds ratio** | | **Willing**  **n (%)** | **OR** | **S.E.** | ***P* value** | **95% C.I. for Odds ratio** | | **Willing**  **n (%)** | **OR** | **S.E.** | ***P* value** | **95% C.I. for Odds ratio** | |
|  |  |  |  |  |  | **Lower** | **Upper** |  |  |  |  | **Lower** | **Upper** |  |  |  |  | **Lower** | **Upper** |
| Description of internet use | Written description | 287 (80.6) | 1 |  |  |  |  | 268 (78.4) | 1 |  |  |  |  | 262 (76.2) | 1 |  |  |  |  |
|  | Pictorial example and written description | 277 (76.3) | 0.715 | 0.258 | .194 | 0.431 | 1.186 | 266 (72.9) | 0.804 | 0.234 | .351 | 0.508 | 1.272 | 278 (73.2) | 1.099 | 0.243 | .698 | 0.683 | 1.769 |
| Gender | Women | 260 (80.2) | 1 |  |  |  |  | 244 (73.9) | 1 |  |  |  |  | 243 (69.8) | 1 |  |  |  |  |
|  | Men | 304 (77.0) | 0.897 | 0.265 | .944 | 0.534 | 1.507 | 290 (76.9) | 1.061 | 0.252 | .815 | 0.647 | 1.740 | 297 (79.0) | 2.218 | 0.263 | .002 | 1.324 | 3.716 |
| Education | Degree level or above | 323 (81.2) | 1 |  |  |  |  | 294 (79.7) | 1 |  |  |  |  | 314 (79.5) | 1 |  |  |  |  |
|  | Basic education | 106 (72.1) | 0.573 | 0.323 | .085 | 0.304 | 1.080 | 104 (72.2) | 0.616 | 0.341 | .155 | 0.316 | 1.210 | 96 (65.8) | 0.682 | 0.353 | .278 | 0.342 | 1.362 |
|  | Advanced education | 135 (77.6) | 0.649 | 0.314 | .168 | 0.351 | 1.201 | 136 (70.1) | 0.606 | 0.284 | .078 | 0.347 | 1.057 | 130 (71.0) | 0.726 | 0.309 | .301 | 0.396 | 1.331 |
| Employ-ment | Employed | 348 (83.5) | 1 |  |  |  |  | 333 (76.7) | 1 |  |  |  |  | 354 (78.3) | 1 |  |  |  |  |
|  | Unemployed | 77 (67.5) | 0.819 | 0.343 | .559 | 0.418 | 1.602 | 64 (73.6) | 1.304 | 0.396 | .503 | 0.600 | 2.835 | 70 (70.0) | 1.151 | 0.387 | .717 | 0.539 | 2.456 |
|  | Retired | 139 (73.9) | 1.243 | 0.392 | .579 | 0.577 | 2.678 | 137 (73.7) | 1.058 | 0.370 | .879 | 0.513 | 2.184 | 116 (67.4) | 1.610 | 0.369 | .197 | 0.781 | 3.317 |
| Income | <£35,000 | 269 (73.7) | 1 |  |  |  |  | 274 (73.1) | 1 |  |  |  |  | 255 (70.6) | 1 |  |  |  |  |
|  | >£35,000 | 295 (83.3) | 1.405 | 0.294 | .248 | 0.789 | 2.500 | 260 (78.3) | 0.802 | 0.278 | .428 | 0.565 | 1.384 | 285 (78.5) | 0.912 | 0.308 | .765 | 0.499 | 1.667 |
| Marital status | Married or in legal partnership | 335 (81.7) | 1 |  |  |  |  | 332 (79.8) | 1 |  |  |  |  | 335 (78.6) | 1 |  |  |  |  |
|  | Single | 144 (72.7) | 1.067 | 0.328 | .844 | 0.561 | 2.029 | 130 (69.9) | 0.591 | 0.291 | .070 | 0.334 | 1.044 | 134 (67.3) | 0.979 | 0.307 | .944 | 0.536 | 1.786 |
|  | Divorced/ widowed/ separated | 85 (76.6) | 1.046 | 0.356 | .900 | 0.520 | 2.102 | 72 (68.6) | 0.667 | 0.353 | .251 | 0.334 | 1.331 | 71 (71.7) | 1.293 | 0.387 | .507 | 0.605 | 2.764 |
| Ethnicity | White | 474 (77.7) | 1 |  |  |  |  | 450 (74.8) | 1 |  |  |  |  | 433 (72.8) | 1 |  |  |  |  |
|  | Asian or Asian British | 23 (67.6) | 0.351 | 0.540 | .052 | 0.122 | 1.011 | 25 (78.1) | 0.585 | 0.565 | .343 | 0.193 | 1.772 | 35 (79.5) | 1.164 | 0.549 | .782 | 0.397 | 3.411 |
|  | Black or Black British | 54 (93.1) | 0.909 | 0.694 | .891 | 0.233 | 3.543 | 50 (84.7) | 0.478 | 0.490 | .132 | 0.183 | 1.248 | 63 (90.0) | 0.838 | 0.558 | .751 | 0.281 | 2.501 |
|  | Mixed or multiple ethnic groups | 13 (76.5) | 0.269 | 0.708 | .064 | 0.067 | 1.078 | 9 (64.3) | 0.420 | 0.727 | .233 | 0.101 | 1.747 | 9 (60.0) | 0.340 | 0.721 | .135 | 0.083 | 1.297 |
| Personal history of condition of interest | No | 456 (77.2) | 1 |  |  |  |  | 436 (75.2) | 1 |  |  |  |  | 371 (72.2) | 1 |  |  |  |  |
|  | Yes | 108 (84.4) | 1.884 | 0.369 | .170 | 0.805 | 3.420 | 98 (77.2) | 1.032 | 0.342 | .928 | 0.528 | 2.016 | 169 (80.5) | 1.478 | 0.302 | .195 | 0.819 | 2.670 |
| Family history of condition of interest | No | 403 (76.9) | 1 |  |  |  |  | 388 (74.6) | 1 |  |  |  |  | 417 (73.8) | 1 |  |  |  |  |
|  | Yes | 161 (82.6) | 1.080 | 0.292 | .793 | 0.609 | 1.915 | 146 (78.1) | 0.898 | 0.288 | .708 | 0.511 | 1.578 | 123 (77.4) | 1.196 | 0.318 | .573 | 0.642 | 2.230 |
| Age | |  | 0.985 | 0.012 | .228 | 0.962 | 1.009 |  | 0.984 | 0.011 | 0.140 | 0.962 | 1.005 |  | 0.981 | 0.011 | .094 | .959 | 1.003 |
| Perceived benefits about sharing internet use data | |  | 7.612 | 0.191 | <.001 | 5.233 | 11.074 |  | 5.706 | 0.165 | <.001 | 4.130 | 7.884 |  | 8.075 | 0.180 | <.001 | 5.675 | 11.488 |
| Perceived concerns about sharing internet use data | |  | 0.452 | 0.191 | <.001 | 0.310 | 0.657 |  | 0.440 | 0.186 | <.001 | 0.306 | 0.633 |  | 0.375 | 0.191 | <.001 | 0.258 | 0.546 |
| Knowledge about internet use data (score) | |  | 1.082 | 0.076 | .299 | 0.932 | 1.257 |  | 1.016 | 0.076 | .834 | 0.876 | 1.179 |  | 0.842 | 0.078 | .027 | 0.723 | 0.981 |
| Number of online services used | |  | 0.943 | 0.052 | .261 | 0.851 | 1.045 |  | 1.046 | 0.047 | .342 | 0.953 | 1.147 |  | 1.018 | 0.051 | .731 | 0.921 | 1.125 |
| Familiarity with internet use data | |  | 1.087 | 0.148 | .575 | 0.813 | 1.453 |  | 0.720 | 0.135 | .015 | 0.552 | 0.938 |  | 0.945 | 0.143 | .706 | 0.706 | 1.266 |

*N included cases: Cancer: 719; heart disease: 707; depression: 724. Nagelkerke’s R: Cancer: .549; heart disease: .517; depression: .582.*

# Definitions of categories for suggestions to increase willingness to share and for concerns

|  | **Category^1^** | **Description** | **Example** |
| --- | --- | --- | --- |
| **What would improve willingness** | No further comments |  | “I would be willing”/ “Nothing really” |
|  | Contribution to research/ society | Survey participants described that the feeling of helping others or contributing to research was sufficient for them to share their internet use data. | “If it helps to diagnose earlier and lead to better treatment it is worth doing” |
|  | Data security assurances | Participants mentioned that general assurances that data is protected would make them more willing to share it. | “If I could be reassured about data protection and that it will not be hacked.” |
|  | Clarification about research purposes and how it works | Participants reported that a clear idea about what the research is about, why it is being conducted, and how it works could increase their willingness to share. | “If there was a well explained argument about how such detection worked and diagnosed this condition.” |
|  | Incentives | Participants mostly described that financial incentives would increase their willingness to share, but some also mentioned better access to treatment or tests as reward for sharing data. | “If I was rewarded/compensated in some way” |
|  | Personal relevance or benefit, e.g., having experienced the condition or getting a quicker diagnosis | Participants reported being more willing to share if they thought they or a family member might be suffering from the target conditions or had experienced this condition themselves or in the family before. | “If I believed I was suffering from depression, even early symptoms” |
|  | Strict governance, personal control and trust | Participants asked for strict policies, laws, and infrastructures or ongoing relationships (requiring these structures) with researchers to increase trust. | “Longer active relationship with the researcher for example if I’d already participated in one or more research activities with them - builds trust.” |
|  | Education and awareness of others and oneself | Participants described how learning more about a condition or research, or educating others about the importance of a condition could increase their willingness to share data. | “To be better educated and gain knowledge.” |
|  | Ease of sharing | Sharing is facilitated if the process of sharing is easy and does not require extra programmes. | “If I didn’t have to download a programme to my computer” |
|  | Proof that it works or concrete examples | Participants reported that positive findings or concrete examples how internet use data can improve early detection would convince them to share their data. | “Positive findings and evidence that this type of research can make a difference in cancer detection and diagnosis.” |
|  | Other, e.g., interest in research and data, embedding data in medical context | Mixed category of rare answers | “Knowing that the search was looked at in context of other factors both personal and external” |
| **Concerns^2^** | No concerns/ further reason not to share |  | “No concerns as of now”/ “Will not share” |
|  | Unspecified concerns | Some participants reported having concerns but did not specify the nature of these concerns. | “Some concerns”/ “Yes, it would worry me” |
|  | Concerns about data security/ privacy | Participants described concerns that their privacy might be invaded or their data might not be stored securely. | “It is a gross invasion of privacy, I would never allow it” |
|  | Data misuse and distrust | Beyond the concern about data privacy, participants were concerned that data might be accessed by third parties who could use the data for marketing purposes, etc. | “I don't trust that you would use my data by selling it onto others who could use if for other nefarious reasons” |
|  | Lack of perceived relevance | Participants were either confused about why their internet data might be relevant for early detection, or convinced it was not relevant. | “My internet usage has absolutely no bearing on the early diagnosis of heart disease” |
|  | Accuracy and misdiagnosis | Participants were concerned that the use of internet data would not be accurate enough to diagnose health conditions, and that there might be a risk of misdiagnosis. | “The accuracy and the potential biases of the diagnosis.” |
|  | Other, e.g., not being judges, using work/ shared devices, causing unnecessary worry | Mixed category of rare answers | “I am using my employers PC, so would not be willing to share search history.” |
